# Vitamin C alleviates aging defects in a stem cell model for Werner syndrome

**DOI:** 10.1007/s13238-016-0278-1

**Published:** 2016-06-06

**Authors:** Ying Li, Weizhou Zhang, Liang Chang, Yan Han, Liang Sun, Xiaojun Gong, Hong Tang, Zunpeng Liu, Huichao Deng, Yanxia Ye, Yu Wang, Jian Li, Jie Qiao, Jing Qu, Weiqi Zhang, Guang-Hui Liu

**Affiliations:** National Laboratory of Biomacromolecules, CAS Center for Excellence in Biomacromolecules Institute of Biophysics, Chinese Academy of Sciences, Beijing, 100101 China; University of Chinese Academy of Sciences, Beijing, 100049 China; State Key Laboratory of Stem Cell and Reproductive Biology, Institute of Zoology, Chinese Academy of Sciences, Beijing, 100101 China; Department of Gynecology and Obstetrics, Peking University Third Hospital, Beijing, 100191 China; Department of Pathology, Carver College of Medicine, University of Iowa, Iowa City, IA 52242 USA; The Key Laboratory of Geriatrics, Beijing Hospital & Beijing Institute of Geriatrics, Ministry of Health, Beijing, 100730 China; FSU-CAS Innovation Institute, Foshan University, Foshan, 528000 China; Beijing Institute for Brain Disorders, Beijing, 100069 China; Department of Pediatrics, Beijing Shijitan Hospital Capital Medical University, Peking University Ninth School of Clinical Medicine, Beijing, 100038 China

**Keywords:** Vitamin C, stem cell, aging, Werner syndrome

## Abstract

Werner syndrome (WS) is a premature aging disorder that mainly affects tissues derived from mesoderm. We have recently developed a novel human WS model using WRN-deficient human mesenchymal stem cells (MSCs). This model recapitulates many phenotypic features of WS. Based on a screen of a number of chemicals, here we found that Vitamin C exerts most efficient rescue for many features in premature aging as shown in WRN-deficient MSCs, including cell growth arrest, increased reactive oxygen species levels, telomere attrition, excessive secretion of inflammatory factors, as well as disorganization of nuclear lamina and heterochromatin. Moreover, Vitamin C restores *in vivo* viability of MSCs in a mouse model. RNA sequencing analysis indicates that Vitamin C alters the expression of a series of genes involved in chromatin condensation, cell cycle regulation, DNA replication, and DNA damage repair pathways in WRN-deficient MSCs. Our results identify Vitamin C as a rejuvenating factor for WS MSCs, which holds the potential of being applied as a novel type of treatment of WS.

**Electronic supplementary material:**

The online version of this article (doi:10.1007/s13238-016-0278-1) contains supplementary material, which is available to authorized users.

## Introduction

Aging is defined as a time-dependent deterioration of organism’s physiological functions that leads to loss of homeostasis and consequently increases susceptibility to morbidity and mortality (Benayoun et al., 2015; Burtner and Kennedy, 2010; Campisi, 2013; Kudlow et al., 2007; Lopez-Otin et al., 2013). Werner Syndrome (referred to as WS, also known as adult progeria) is a premature aging disorder with phenotypes such as grey hair, osteoporosis, diabetes, and cancer. WS is caused by mutations in the *WRN* gene, which is involved in several fundamental cellular mechanisms, including DNA replication, DNA repair, and telomere maintenance (Burtner and Kennedy, 2010; Kudlow et al., 2007; Lopez-Otin et al., 2013). Since the expression of WRN also decreases during physiological aging (Polosak et al., 2011; Zhang et al., 2015), WS may be a relevant model for studying physiological aging and aging-associated disorders (Burtner and Kennedy, 2010; Kudlow et al., 2007; Lopez-Otin et al., 2013).

The advances in pluripotent stem cell and gene editing techniques have opened a new avenue to study the pathogenesis of human premature aging syndromes and aging-related diseases (Fu et al., 2016; Liu et al., 2011; Liu et al., 2012b; Liu et al., 2014; Lo Cicero and Nissan, 2015; Miller et al., 2013; Pan et al., 2016; Zhang et al., 2015). They also provide a powerful platform for drug screening and validation of their efficacy (Blondel et al., 2016; Liu et al., 2012a; Liu et al., 2012b; Liu et al., 2014; Yang et al., 2014; Zhang et al., 2013). We have recently developed a human stem cell model by homozygous depletion of the exons 15 and 16 of *WRN* alleles, which recapitulates the major cellular defects of WS, including accelerated senescence, growth arrest, telomere attrition, increased DNA damage response, excessive production of inflammatory factors, as well as increased stem cell attrition in the *in vivo* niche (Zhang et al., 2015). We also identify heterochromatin disorganization as a driver for WS MSC aging, and overexpression of heterochromatin component HP1α can partially rescue the accelerated aging defects in the WS MSCs (Zhang et al., 2015). These findings suggest that epigenetic alterations could underlie human cellular aging, and the “epigenetic aging” can be repressed or reversed under specific context. It is unknown, however, if the premature aging processes can be alleviated by chemicals or drugs.

Here, utilizing the WS MSC model, we tested the potential rescuing effect with a group of compounds which have been reported with “anti-aging” or “longevity-promoting” activity from different model organisms. Among them, Vitamin C (VC, also known as ascorbic acid) showed the best efficacy on alleviation of the aging defects in WS MSCs.

## Results

### Screening for chemicals capable of repressing accelerated cellular senescence in WS MSCs

**Figure 1:**
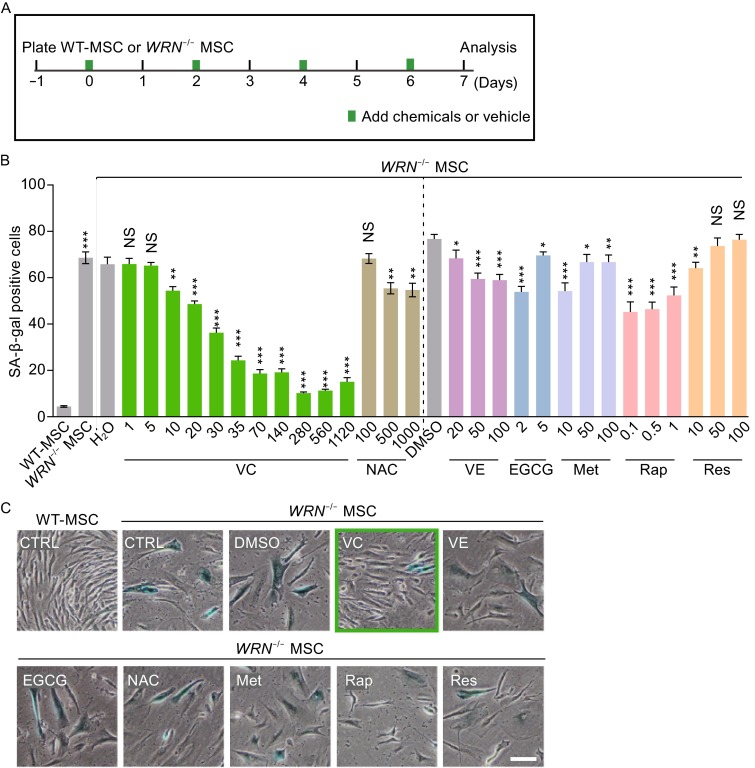
Chemicals screening for alleviating premature aging in WS MSCs. (A) Schematic demonstration of the chemical screening protocol. 3 × 10^4^ wild-type (WT) or *WRN* ^-/-^ MSCs (P5) were seeded in one well of 6-well dish, following with the treatment with the chemicals or vehicle control (water or DMSO) and refreshed every other day. Cell senescence, assayed by senescence associated β-galactosidase (SA-β-gal) staining, was analyzed 1 week later. (B) Frequency of SA-β-gal positive cells in WT or *WRN* ^-/-^ MSCs with or without chemical treatment. For each molecule, different concentrations were used as indicated along the X axis. Data are represented as mean ± SEM, *P < 0.05, **P < 0.01, ***P < 0.001, NS, not significant by *t* test; *n* ≥ 3. (C) Representative images of SA-β-gal staining. VC: 280 µmol/L; VE: 20 µmol/L; EGCG: 2 µmol/L; NAC: 100 µmol/L; Met: 10 µmol/L; Rap: 0.1 µmol/L; Res: 10 µmol/L. Scale bar, 100 µm

Using our recently established WS MSC aging model (Zhang et al., 2015), we have screened a panel of known anti-oxidants and other chemicals with reported anti-aging effects, including VC, Vitamin E (VE), (-)-epigallocatechin gallate (EGCG), N-Actyl-L-cysteine (NAC), Metformin (Met), Rapamycin (Rap), and Resveratrol (Res) (Baur et al., 2006; Cao et al., 2011; Dallaire et al., 2014; Harrison et al., 2009; La Fata et al., 2014; Lebel et al., 2010; Martin-Montalvo et al., 2013; Na et al., 2008). We designed an *in vitro* screening platform by using late passage (passage 5) of *WRN*^-/-^ MSCs at low confluence. The low cell density allowed for evaluating stringent phenotypic rescue and maximizing the efficacy of chemicals. We treated the cells with the indicated chemicals up to one week (Fig. 1A). Among all the chemicals included, we identified VC as the most potent agent that significantly reduced the frequency of senescence-associated-β-galactosidase (SA-β-gal) positive cells in a dose-dependent manner (Fig. 1B and 1C). The efficacy of VC on suppressing senescence was very prominent at physiological concentrations starting from as low as 10 µmol/L, relative to the vehicle treatment group. At the proposed physiological doses of plasma VC (Du et al., 2012), ranging from 35 to 70 µmol/L, VC exhibited close to the optimal suppressive effect on senescence (compared between 68% in control group, 20%–23% in physiological dosages (35–70 µmol/L), and 10% in optimal dose (280–560 µmol/L) (Fig. 1B). Other compounds, including VE, EGCG, NAC, Met, Rap, and Res also showed mild activity in reducing SA-β-gal positive subpopulation (Fig. 1B and 1C), but the effects were not as significant as VC’s. These results indicate that VC has a unique activity in repressing accelerated senescence in WS MSCs.

### VC suppresses aging-related parameters

**Figure 2:**
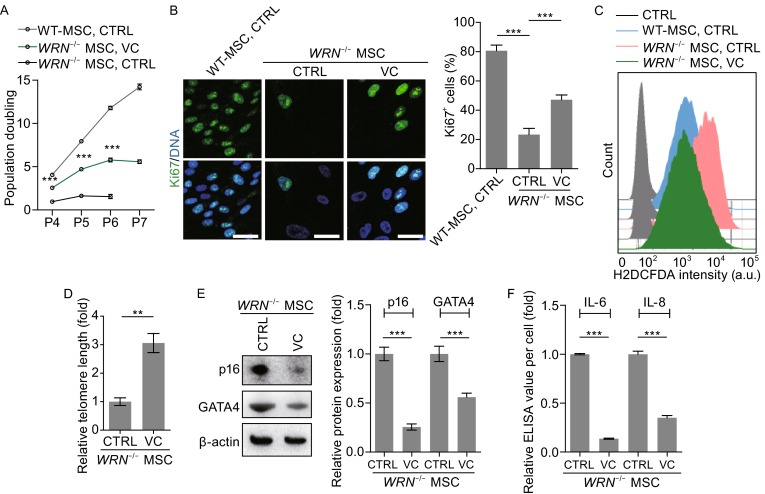
VC represses aging properties in WS MSCs. (A) Growth curve analyzing the population doubling of MSCs. (B) VC promoted proliferation in *WRN* ^-/-^ MSCs. Representative immunofluorescence staining (left) and quantitative analysis (right) of Ki67 in vehicle or VC treated (7 days) WT MSCs and *WRN* ^-/-^ MSCs. Scale bar, 50 µm. (C) H2DCFDA based measurement of reactive oxygen species (ROS) in vehicle or VC treated (7 days) WT MSCs and *WRN* ^-/-^ MSCs. (D) Telomere lengths of WS MSCs with or without VC treatment were measured by quantitative RT-PCR. (E) Expression of senescence-associated proteins p16^Ink4a^ and GATA4 was examined by Western blot, and quantitative results were shown on the right. (F) ELISA showing a decrease in IL-6 and IL-8 secretion in *WRN* ^-/-^ MSCs after VC treatment. The values were normalized by the cell numbers. Data are represented as mean ± SEM, **P < 0.01, ***P < 0.001; *n* ≥ 3

We have shown that the WS MSCs exhibited many features of premature cellular senescence, such as decreased proliferation, elevated senescence-associated secretory phenotype (SASP), and heterochromatin alterations etc. (Zhang et al., 2015). To determine the impact of VC on these parameters, we treated WS MSCs with VC and examined different cellular properties related to aging. Consistent with the reduced premature cellular senescence, VC treatment reactivated the cellular proliferation potential and upregulated the frequency of Ki67 positive cells (Fig. 2A and 2B). Reactive oxygen species (ROS) production, as indicated by the positive staining of H2DCFDA, was significantly elevated in the senescent WS MSCs as previously reported for WS mouse fibroblasts (Labbe et al., 2010), which was reduced to a similar level as in the WT MSC cells by VC treatment (Fig. 2C). In addition, treatment of WS MSCs with VC repressed accelerated telomere shortening (Fig. 2D) (Zhang et al., 2015), down-regulated expression of aging markers, such as p16^Ink4a^ and GATA4 (Fig. 2E) (Kang et al., 2015), and effectively alleviated SASP, including the production of pro-inflammatory cytokines such as IL-6 and IL-8 (Fig. 2F).

**Figure 3:**
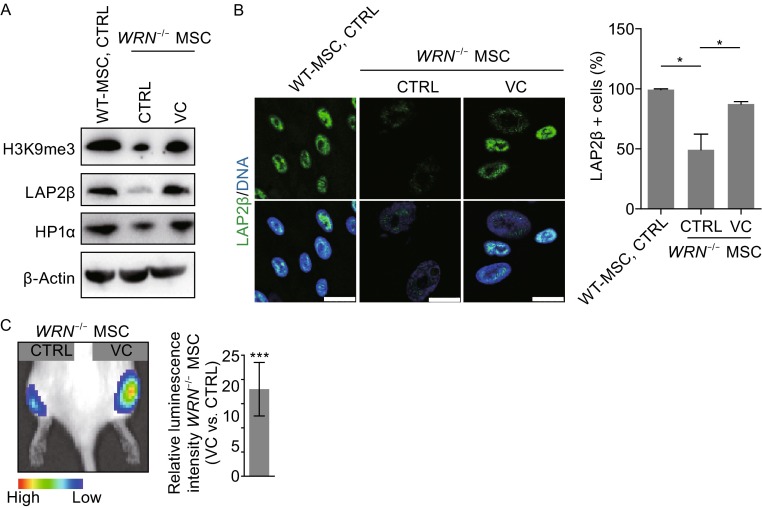
VC restores epigenetic parameters and *in vivo* viability of WS MSCs. (A) Western blot analysis of the indicated proteins in MSCs. (B) VC increased heterochromatin markers by immunofluorescence staining. Representative immunofluorescence staining (left) and quantitative analysis (right) of LAP2β in vehicle or VC treated (7 days) WT MSCs and *WRN* ^-/-^ MSCs. Scale bar, 25 µm. (C) Luciferase activity of WS MSCs was detected by *in vivo* imaging system (IVIS) one week after implantation, and quantitative results were shown on the right. All data are represented as mean ± SEM. *P < 0.05, ***P < 0.001 by *t* test; *n* ≥ 3

Heterochromatin alteration is one of the hallmarks and is a driver for WS MSC aging (Lopez-Otin et al., 2013; Zhang et al., 2015). Western blotting showed that levels of heterochromatin markers HP1α and H3K9me3 were up-regulated upon VC treatment, indicating that VC promotes remodeling of heterochromatin to a younger state. In line with the heterochromatin changes, the expression of LAP2β, the heterochromatin-anchoring inner nuclear membrane protein, was also elevated (Fig. 3A and 3B). In addition, we found an increase in the number of nuclear foci for γ-H2AX and phosphorylated ATM/ATR substrates in WRN-deficient MSCs; VC had no influence on these foci formation (Fig. S1A), suggesting a possibility that the restoration of heterochromatin and nuclear lamina components may not be associated with alleviation of DNA damage response (DDR) in WS MSCs. Together, these results indicate that VC is able to rejuvenate the heterochromatin and nuclear lamina architectures in WS MSCs, a process independent of the DNA damage response.

To investigate whether VC can restore the MSC’s *in vivo* activity, luciferase-labeled WS MSCs were pre-treated with VC, and implanted into the tibialis anterior muscle of the immunodeficient mice, and then engraftment and survival were determined by measuring luminescence signals after 7 days. In line with the observed repression of accelerated cellular decay *in vitro*, VC treatment effectively restored the *in vivo* viability of WS MSCs (Fig. 3C).

### VC inhibits aging related genes and pathways in the WS MSC model

**Figure 4:**
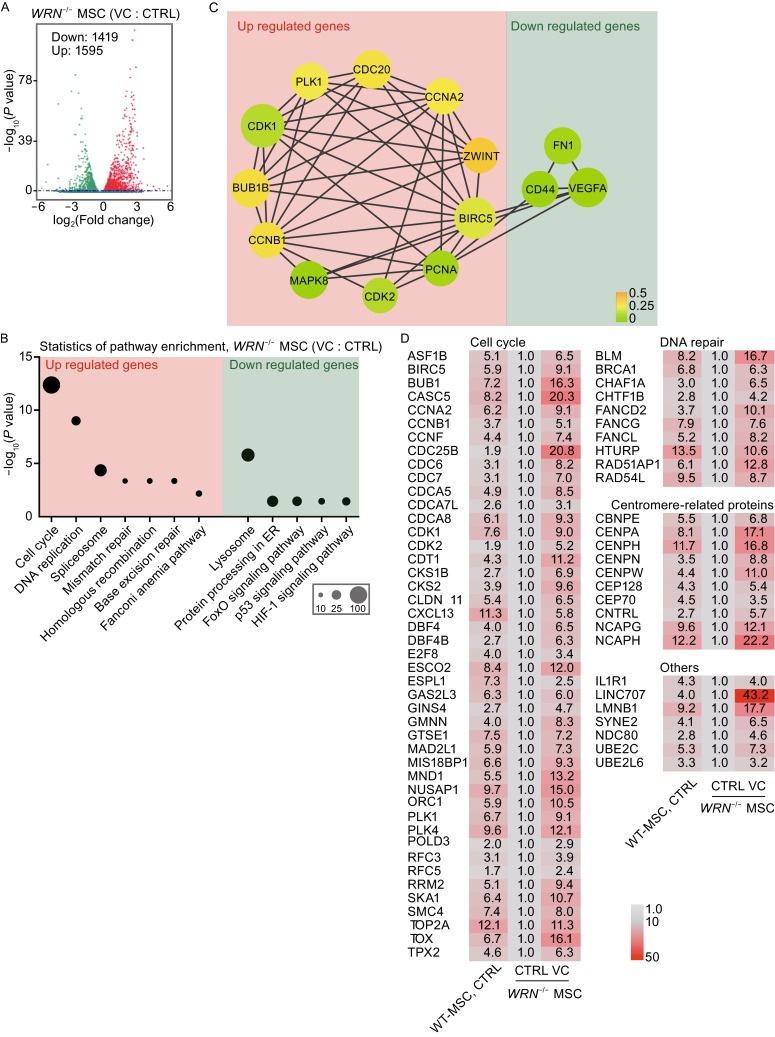
VC induced a global transcriptome change of aging-suppressing genes and pathways. (A) Volcano plot showing significantly altered genes (|log_2_(Fold change)| > 1, *P* value < 0.05) between vehicle- and VC-treated *WRN* ^-/-^ MSCs. FC, fold change. (B) KEGG based pathway enrichment analysis of the significantly altered gene sets (green: down regulated genes; red: up regulated genes) in *WRN* ^-/-^ MSCs upon VC supplement. Number of altered genes in each pathway was indicated by size of the bubble. (C) Predicted protein-protein interactions (PPI) analysis of differentially expressed genes was based on the STRING database, according to e-value = 1 × 10^−10^ and string score > 700, differentially expressing genes with interaction frequency > 80 were illustrated on the picture. Node size indicated the degree of interaction and node color indicated clustering co-efficiency (green: down regulated genes; red: up regulated genes). (D) Heatmap of mRNA levels between vehicle- and VC-treated *WRN* ^-/-^ MSCs

To uncover the molecular mechanism underlying how VC rejuvenates WS MSCs, we performed genome-wide RNA sequencing (RNA-seq). We identified 1595 upregulated genes and 1419 downregulated genes (|log_2_(Fold change)| > 1, *P* value < 0.05) in VC treated WS MSCs relative to vehicle treated cells (Fig. 4A, S1B–C and Supplementary tables). Gene Ontology (GO) Term analysis for cellular component, biological pathways and molecular functions indicated that the most significant pathway (*P* value = 0.00036) for the VC-upregulated genes in WS MSCs was “chromosome organization” (Fig. S1D), which is also the most significant GO term for downregulated genes in WS MSCs compared to wild-type MSCs (Zhang et al., 2015). Notably, VC induced the re-expression of chromosome-packaging proteins at centromeres in WS MSCs, which is consistent with a rescue of cellular aging process (Fig. S1D) (Zhang et al., 2015).

We also analyzed the differentially expressed genes using KEGG database. We found “cell cycle” as the top remarkable set of genes upregulated by VC, in agreement with the increased Ki67 staining as the proliferation index (Fig. 3B). Consistently, the transcripts for many of these cell cycle genes are also the ones we initially identified to be down-regulated in WS MSCs (Zhang et al., 2015). We noticed that the molecular network for cell cycle entry was partially re-activated by VC (Fig. 4B and 4C). Of note, the cell mitosis-promoting proteins such as PCNA, CDK1, and CDK2 were also significantly upregulated in VC-treated WS MSCs (Fig. 4C and 4D). In addition, we also found that VC was involved in DNA replication and repair pathways. For example, VC treatment led to the reactivation of the DNA replication pathway that was repressed in WRN-deficient MSCs (Fig. 4B) (Zhang et al., 2015), and of the various DNA repair pathways including mismatch repair, homologous recombination, base excision repair, and Fanconi anemia pathway (Zhang et al., 2015) (Fig. 4B). On the other hand, VC down-regulated several signaling pathways that are normally activated during cellular senescence, such as P53, FoxO, and HIF-1 pathways (Lopez-Otin et al., 2013; Martins et al., 2015), as well as the pathways related to lysosome degradation and protein-processing in endoplasmic reticulum (ER) (Fig. 4B). These changes were validated by quantitative RT-PCR (Fig. 4D). Together, these results clearly indicate that VC plays a role in re-activation of aging-suppressing genes and related signaling pathways at the transcriptional level.

## Discussion

The combination of gene editing technique and human pluripotent stem cells (hPSC) including induced pluripotent stem cells (iPSCs) and embryonic stem cells offers a new way to model genetic diseases for studying disease-causing mechanisms and screening drugs *in vitro*. Since 2011, we and others have shown the capacity of hPSC disease models in recapitulating disease defects in various human genetic disorders including Hutchinson-Gliford progeria syndrome (HGPS), Parkinson's diseases, Fanconi Anemia, xeroderma pigmentosum, glioblastoma, and WS (Cheung et al., 2014; Duan et al., 2015; Fu et al., 2016; Liu et al., 2011; Liu et al., 2012a; Liu et al., 2012b; Liu et al., 2014; Park et al., 2008; Saha and Jaenisch, 2009; Yu et al., 2013; Zhang et al., 2015). We have also provided evidence showing that these disease models could be used to examine the functional effects of chemical compounds as well as to re-purpose the old drugs already approved for different diseases in clinics. In this study, we identify VC as a potent agent to alleviate aging process in a stem cell model for adult progeria. Among the compounds we tested, VC has the most significant effect on repressing cellular senescence (indicated by SA-β-gal staining) and on promoting the WS MSC growth. For example, whereas the mTOR inhibitor rapamycin has been reported to improve the proliferation potential in HGPS fibroblasts (Cao et al., 2011), it showed marked repressive effect on the self-renewal of WS MSCs (Fig. 1B, 1C and data not shown). VC effectively represses the accelerated cellular senescence, improves the stem cell self-renewal, decreases SASP in WS MSCs, and alleviates telomere attrition. This finding is of great interest because previous studies on *C. elegans* and mouse models have showed that a continuous long-term treatment of WS individuals with high doses of VC is a promising therapeutic approach for this syndrome (Dallaire et al., 2014; Lebel et al., 2010). Our study, together with evidences from *C. elegans* and mouse models, provides an important cue to WS therapy. Recent study indicated that the blood of WS patient has increased level of IL-6 (Davis and Kipling, 2006). Consistently, our WS stem cell model recapitulated the upregulation of IL-6, a phenotype of SASP, in the medium of WS MSC cultures (Zhang et al., 2015). We also identify that VC decreases the IL-6 secretion in WS MSCs. This suggests a possibility of employing VC as a beneficial factor to decrease the inflammatory mediators for WS patients. Given that VC restores the *in vivo* viability of WS MSC, one may expect that VC could help to eliminate the harmful effects of senescent cells and/or counteract the premature stem cell deterioration *in vivo* (Baker et al., 2016; Baker et al., 2011). Although human unlike rodents cannot synthesize VC in the body, the water-soluble property of VC makes it very easy to be supplied through dietary sources and supplements. Our study shows that high concentrations of VC (i.e. 280 µmol/L) produce the best effect on rejuvenating WS MSCs without discernible cytotoxicity (Du et al., 2012). Oral supplement of VC normally reaches micromolar level in the plasma, with the highest plasma concentration around 80 µmol/L, which is the suboptimal dose for alleviating the aging process. However, in clinical trials of cancer therapy, intravenous injection of VC can increase the plasma level of VC up to millimolar levels (Du et al., 2012; Fukushima and Yamazaki, 2010). Thus, it would be of particular interest to titrate the optimal dosage of VC used in clinics from which WS patients will benefit mostly. One concern is that higher level of VC tends to induce free radicals, a phenotypic switch from anti-oxidant to oxidizing agent (Du et al., 2012). Thus the titration for optimal VC concentration in human patients needs to be very cautiously performed.

Regarding the molecular mechanism, we found that treatment with VC effectively diminishes cellular ROS in WS MSCs. This could be one of the molecular mechanisms underlying VC’s effect, given that WS MSCs exhibit much higher ROS levels than their wild-type counterparts. We found that, besides VC, other ROS eliminators also show an effect on the rejuvenation of WS MSCs. For example, NAC, a strong antioxidant, and Met and EGCG, activators of NRF2 antioxidant pathway (Martin-Montalvo et al., 2013; Na et al., 2008), all alleviated the aging defects of WS MSCs. One would postulate that an increased anti-oxidizing capability in WS MSCs could principally counteract the harmful effect of ROS on compromising the biomacromolecule machinery (i.e. DNA or protein) inside the cells. On the other hand, elevated ROS levels have recently been known as a factor leading to heterochromatin disorganization (Frost et al., 2014). In this context, reduction of ROS levels may provide an explanation for restoration of heterochromatin architecture by VC. However, it should also be noted that NAC, a much stronger antioxidant, only show mild rescue activity towards WS MSCs. Therefore, this raises a possibility that VC may also exert an effect in a ROS-scavenger independent manner. Recent evidence points to a more direct role of VC in regulating epigenetic reprogramming (Young et al., 2015). For instance, VC functions as a cofactor for TET dioxygenases that catalyze the oxidation of 5-methylcytosine (5mC) to 5-hydroxymethylcytosine (5hmC), further resulting in the generation of 5-formylcytosine (5fC), 5-carboxylcytosine (5caC), and unmodified cytosine. In addition, VC serves as a cofactor of the JumonjiC (JmjC)-domain containing histone demethylases (i.e. JHDM1) (Chen et al., 2013; Esteban and Pei, 2012; Esteban et al., 2010; Pera, 2013; Wang et al., 2011; Yulin et al., 2012). A more direct evidence is that VC promotes proliferation of bone marrow-derived MSCs and facilitates the derivation of iPSCs from MSCs (Yulin et al., 2012). How VC coordinates different mechanisms in alleviating aging defects in WS MSCs warrants further investigation.

**Figure 5:**
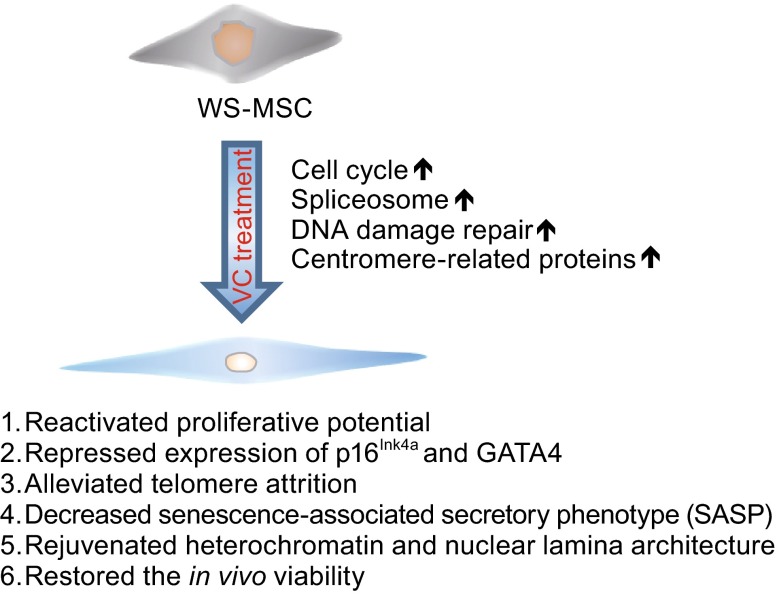
A proposed model illustrating that VC represses aging properties in WS MSCs by causing a global transcriptome change of aging-suppressing genes and pathways

In addition, consistent with a role of VC in rejuvenating the nuclear lamina and heterochromatin in WS MSCs, our study revealed that VC reset the gene expression profiles of WS MSCs to a younger state. Our recent study has indicated that a group of genes linked to chromatin condensation, cell cycle, DNA replication, and DNA repair were all repressed in WS MSCs (Zhang et al., 2015). Intriguingly, parts of these genes were reactivated by VC treatment in WS MSCs. These observations verified that VC has the capability to help WS MSCs to reprogram the senescent transcriptome probably by reactivating the stemness-associated core transcriptional network. Moreover, our study also demonstrate that VC treatment can downregulate pro-senescence pathways, including P53, FoXO, and HIF etc., which may provide another layer of mechanisms in recovering young MSC phenotypes (Fig. 5).

## Materials and methods

### Cell lines generation, culture and treatment

WRN gene knockout human ESC clones (referred to as *WRN*^-/-^-ESC) were generated as previously described (Zhang et al., 2015). Differentiation, purification, culture, and characterization of WT-ESC and *WRN*^-/-^-ESC derived mesenchymal stem cells (MSCs) were performed as described previously (Zhang et al., 2015).

Procedure of cell treatments with chemicals at indicated concentrations was illustrated in Fig. 1A, including Vitamin C (VC, Sigma, A4403), (-)-epigallocatechin gallate (EGCG, Sigma, E4143), Vitamin E (VE, Sigma, T1782), N-Actyl-L-cysteine (NAC, Sigma, A7250), Metformin (Met, Tocris, 2864), Rapamycin (Rap, Tocris, 1292), and Resveratrol (Res, Sigma, R5010). VC and NAC were dissolved in water. All other compounds were dissolved in DMSO. Final concentrations of DMSO did not exceed 0.1%.

### Reagents

Antibodies were purchased from the following companies. Abcam: anti-H3K9me3 (ab8898); Santa Cruz Biotechnology: anti-β-actin (SC-130301), anti-GATA4 (SC-1237); Cell Signaling Technology: Phospho-(Ser/Thr) ATM/ATR Substrate Antibody (2851), anti-HP1α (2616), anti-p16 (4828); Millipore: anti-γ-H2AX (05-636); BD Bioscience: anti-LAP2β (611000); Vector: anti-Ki67 (VP-RM04).

### Senescence associated β-galactosidase (SA-β-gal) staining

SA-β-gal staining was performed as described previously (Debacq-Chainiaux et al., 2009). Briefly, cultured cells were washed in PBS and fixed at room temperature for 3 min in 2% formaldehyde and 0.2% glutaraldehyde. Fixed cells were stained with fresh staining solution for SA-β-gal activity at 37°C overnight, and counted for positivity (N > 300).

### ROS detection

Oxidative stress levels were quantified through H2-DCFDA (Invitrogen, C6827) based flow cytometry following manufacturer’s instructions

### ELISA

Kits to detect IL-6 (D6050) and IL-8 (D8000C) were purchased from R&D Systems, and used as previously reported (Zhang et al., 2015). All ELISA data were normalized to cell numbers

### Population doubling time (PDT)

Cell numbers of each passage were counted by hemocytometer after trypan blue staining, and then PDT was calculated according to previous formulae (Zhang et al., 2015)

### Western bloting and quantitative RT-PCR

For Western blot, preparation of lysate from cell culture was described as previously reported. BCA kit purchased from Thermo Fisher Scientific was used for protein quantification. Equal amounts of protein lysates were subjected to the wells of the SDS-PAGE gel, and then electrotransferred to a PVDF membrane. After blocking, the membrane was incubated with appropriate dilutions of primary antibody and secondary antibodies (Jackson ImmunoResearch Labs). Then imaging was performed using ChemiDoc XRS system (Bio-Rad).

For quantitative analysis of gene expression, total RNA was extracted according to previous protocol (Zhang et al., 2015). Then cDNA was synthesized by GoScript™ Reverse Transcription System (Promega), followed by removal of genomic DNA with DNA-free™ Kit from Ambion. Quantitative RT-PCR was performed using SYBR® Green Supermix (TOYOBO). Quantitative PCR-based method was used to measure telomere length according to established protocol (Zhang et al., 2015). Primer sequences are listed in Table S1.

### Immunofluorescence microscopy

Cells were washed once with PBS and fixed in 4% formaldehyde at room temperature. Subsequently, cells were blocked with 10% donkey serum and 0.1% Triton X-100 in PBS for 1 h, and then diluted primary antibodies were added and incubated at 4°C overnight. After three consecutive washes in wash buffer, cells were incubated for 45 min with secondary antibodies (Alexa Fluor Donkey-anti-mouse and Alexa Fluor Donkey-anti-rabbit, Invitrogen) together with Hoechst 33342 (Invitrogen). After washed, samples were covered with VECTASHIELD mounting medium (Vector) and imaged in Leica SP5 confocal. Acquisition parameter was same for each experiment. Around 100 randomly selected cells were analyzed.

### *In vivo* cell viability analysis

MSC implantation was performed as previously described (Zhang et al., 2015). In brief, 5 × 10^5^ luciferase-expressing WS MSCs were pretreated with vehicle or 280 μmol/L VC for one week and then implanted into the middle of the tibialis anterior muscle of immunodeficient mice. Seven days after implantation, mice were anaesthetized and injected with D-luciferin solution. Fifteen minutes later, *in vivo* luciferase activity of each mouse was determined by the IVIS lumina system (PerkinElmer). Luminescence intensity was normalized to luciferase intensity of MSCs just before implantation. Animal experiments performed in this study were approved by the Institute of Biophysics, Chinese Academy of Science.

### RNA-seq library construction

Two million cells were applied to extract total RNA as previously described (Zhang et al., 2015). RNA integrity was assessed using the RNA Nano 6000 Assay Kit of the Bioanalyzer 2100 system (Agilent Technologies). A total amount of 3 μg RNA per sample was used as input material. For the RNA sample preparations, sequencing libraries were generated using NEBNext® Ultra™ RNA Library Prep Kit for Illumina® (NEB) following manufacturer’s recommendations and index codes were added to attribute sequences to each sample. Then, PCR products were purified (AMPure XP system) and library quality was assessed using the Agilent Bioanalyzer 2100 system. At last, the library preparations were sequenced on an Illumina Hiseq platform and 125 bp/150 bp paired-end reads were generated.

### RNA-seq data analysis

Then reads were mapped to the human reference genome hg19 (from UCSC) by TopHat v2.0.12. Transcript expression and differentially expressed genes were analyzed as previously reported (Wu et al., 2015). Briefly, HTSeq v0.6.1 was used to count the reads numbers mapped to each gene. And then FPKM (Fragments per kilobase of transcript sequence per millions) of each gene was calculated based on the length of the gene and reads count mapped to this gene. Differential expression analysis of two groups (two biological replicates per condition) was performed using the DESeq R package (1.18.0). Gene Ontology (GO) enrichment analysis of differentially expressed genes was implemented by the GOseq R package, in which gene length bias was corrected. KOBAS software was used to test the statistical enrichment of differential expression genes in KEGG pathways. GO terms and pathway enrichment with corrected *P* value less than 0.05 were considered significantly enriched by differential expressed genes. Protein-protein interactions (PPI) prediction of differentially expressed genes was based on the STRING database.

## Electronic supplementary material

Below is the link to the electronic supplementary material.

Supplementary material 1 (DOCX 1319 kb)
