# Supplementary material for: Vitamin C alleviates aging defects in a stem cell model for Werner syndrome
Source: Protein Cell. 2016 Jun 6;7(7):478–88. doi: 10.1007/s13238-016-0278-1 (PMC4930768; doi:10.1007/s13238-016-0278-1)
Supplement: Supplementary file 1 — Supplementary material 1 (DOCX 1319 kb) [file 13238_2016_278_MOESM1_ESM.docx]

**Supplementary information**

**
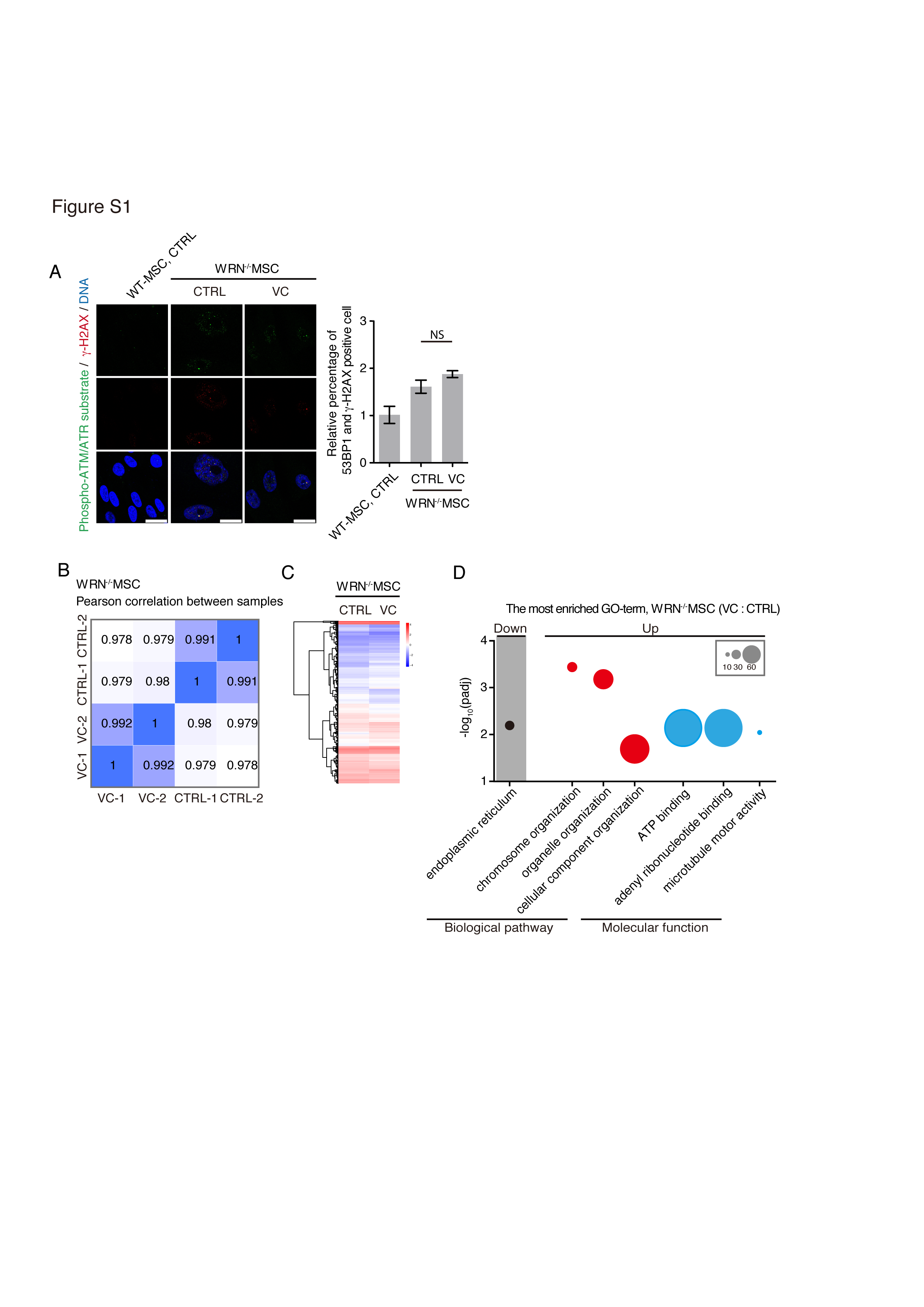
**

**Figure S1. (A)** Representative immunofluorescence staining and quantitative analysis of indicated proteins in vehicle- or VC-treated (7 days) WT MSCs and *WRN*^-/-^ MSCs. Scale bar, 25 μm. **(B)** Heatmap showing the correlation between the biological replicates. **(D)** Heatmap showing significantly altered gene (P-value < 0.05, FC[VC/vehicle] < 0.5 or FC [VC/vehicle] > 2) between VC- and vehicle-treated *WRN*^-/-^ MSCs. FC, fold change. **(E)** GO analysis of the significantly alter genes (Down: down regulated genes; Up: up regulated genes) in MSCs upon VC exposure. Number of altered genes in each term was indicated by size of the bubble. NS means not significant by t test; n=3.

**Supplementary table S1**

**Quantitative RT-PCR primers used in this study**

| Gene | Forward primer (5’->3’) | Reverse primer (5’->3’) |
| --- | --- | --- |
| 18S | GTAACCCGTTGAACCCCATT | CCATCCAATCGGTAGTAGCG |
| ASF1B | TCCGGTTCGAGATCAGCTTC | GTCGGCCTGAAAGACAAACA |
| BIRC5 | AGGACCACCGCATCTCTACAT | AAGTCTGGCTCGTTCTCAGTG |
| BUB1 | AAATGACCCTCTGGATGTTTGG | GCATAAACGCCCTAATTTAAGCC |
| CASC5 | CTTCACACCGAGGACTCAAGA | TTTGATGTGTAGAAGAGGCACTG |
| CCNA2 | CGCTGGCGGTACTGAAGTC | GAGGAACGGTGACATGCTCAT |
| CCNB1 | TCGCATCAAACTCTCTGGCTA | TGAGCGACTAAACTCACCACT |
| CCNF | CACAAAGCATCCATATTGCACTG | TGGTCAGACATCCCTGATGAG |
| CDC25B | ACGCACCTATCCCTGTCTC | CTGGAAGCGTCTGATGGCAA |
| CDC6 | ATGGCACAGTATCTGGAGGAG | TAAGCTGGACTCACTCTCGGA |
| CDC7 | AGGGTAGATGCGGTTACTATGT | AGCGTGTTCACCACAAAATCTT |
| CDCA5 | GAGGTCCCAGCGGAAATCAG | TCTTTAAGACGATGGGCTTTCTG |
| CDCA7L | TTGGTCTTCGAGTAGCCTTTCA | CTCATCCCGAGAGTCATCCTC |
| CDCA8 | GAAGGGCAGTAGTCGGGTG | TCACGGTCGAAGTCTTTCAGA |
| CDK1 | CACCAGCGTTATATTTTGCACAG | ATCTGCCAGCTTTAACTCCCC |
| CDK2 | TGTTTAACGACTTTGGACCGC | CCATCTCCTCTATGACTGACAGC |
| CDT1 | CGGTGGACGAGGTTTCCAG | CTGCCGGGGTGGATTTCTT |
| CKS1B | TATTCGGACAAATACGACGACG | CGCCAAGATTCCTCCATTCAGA |
| CKS2 | TTCGACGAACACTACGAGTACC | GGACACCAAGTCTCCTCCAC |
| CLDN11 | CGGTGTGGCTAAGTACAGGC | CGCAGTGTAGTAGAAACGGTTTT |
| CXCL13 | GCTTGAGGTGTAGATGTGTCC | CCCACGGGGCAAGATTTGAA |
| DBF4 | CCGGAAAGTCCTTTTACTTGGAT | AACCCTCAATTACCCCACCCA |
| DBF4B | CCGGAAAGTCCTTTTACTTGGAT | AACCCTCAATTACCCCACCCA |
| E2F8 | ATCTGCCTTGACGAAGTGGC | GGCGTACTTATTCTCCTCCCC |
| ESCO2 | CACTGGGACGCACCCAAAA | CACTTGCCTTGTCGCAAAAG |
| ESPL1 | CCGCCTTGAAGGAGTTCCTG | GGGGTAGACACTAAGTAGCCAT |
| GAS2L3 | AGCCTGCAATTCAAGTATGGTT | TGGTCCGTGTCTGGGAGTC |
| GINS4 | AGTTGGCCTTTGCCAGAGAG | GAACTGCCCGAAAGAGGTCC |
| GMNN | GCCCTGGGGTTATTGTCCC | AGCGCCTTTCTCCGTTTTTCT |
| GTSE1 | CAGGGGACGTGAACATGGATG | ATGTCCAAAGGGTCCGAAGAA |
| MAD2L1 | GAGAAGTCCGAAGAAACTCACG | CCGAAGCGTTGAGAGGTTCC |
| MIS18BP | CAGGCACACTTACTCCTGTAAAA | GAGGTAGTAGCCTCTGTTAGCA |
| MND1 | CAGTGCCGAAGAATCGGTCAT | CGAGCAGCATTTAATGGAGACA |
| NUSAP1 | AGCCCATCAATAAGGGAGGG | ACCTGACACCCGTTTTAGCTG |
| ORC1 | ACTACCCCACAAGGCTGAAGA | AGTGCAGTTTTCGATCCAACA |
| PLK1 | CAGTCACTCTCCGCGACAC | GAGTAGCCGAATTGCTGCTG |
| PLK4 | AAGCTCGACACTTCATGCACC | GCATTTTCAGTTGAGTTGCCAG |
| POLD3 | GAGTTCGTCACGGACCAAAAC | GCCAGACACCAAGTAGGTAAC |
| RFC3 | GTGGACAAGTATCGGCCCTG | TGATGGTCCGTACACTAACAGAT |
| RFC5 | GAAGCAGACGCCATGACTCAG | GACCGAACCGAAACCTCGT |
| RRM2 | TGGTGAAGCGGCCTAATCC | GCAACATGAGTCGAAAGGTCG |
| SKA1 | CCTGAACCCGTAAAGAAGCCT | TCATGTACGAAGGAACACCATTG |
| SMC4 | CCGCTGGTCATCGGACAATTT | GCGCACATAATAGTTGGAGCCT |
| TOP2A | ACCATTGCAGCCTGTAAATGA | GGGCGGAGCAAAATATGTTCC |
| TOX | TGGTTGGGTGGTCGTCAAATC | CAGACGCAATTCCTCCACAGT |
| TPX2 | ATGGAACTGGAGGGCTTTTTC | TGTTGTCAACTGGTTTCAAAGGT |
| BLM | CAGACTCCGAAGGAAGTTGTATG | TTTGGGGTGGTGTAACAAATGAT |
| BRCA1 | GCTCGTGGAAGATTTCGGTGT | TCATCAATCACGGACGTATCATC |
| CHAF1A | AGCCCGTCTGCCGTTTAAG | AGAAGTACCCTGATCGTCTGAC |
| CHAF1B | CTGGCGTGAGAAGCAAAGA | CTGGCGTGAGAAGCAAAGA |
| FANCD2 | AAAACGGGAGAGAGTCAGAATCA | ACGCTCACAAGACAAAAGGCA |
| FANCG | AATGACCGGCTCGTTCGAC | CTTGCCCTCAGGATAATGAAGTT |
| FANCL | AGAGCTGTATGCACTACCTCC | ATCCCAACCAAGAGTTCCTATCT |
| HJURP | CACAAAGCCATCAAGCATCATC | TCAGAGCAGGGTATGAAGTTCT |
| RAD51AP | AAGTCTGGGAGTTGTCCCCTA | ACTGCAAACAGCTACTCATTTGG |
| RAD54L | AGGCAGGTCCTGTGATGATGA | TCAAAGGTTTCCGAAAAGGAGAC |
| CENPE | GATTCTGCCATACAAGGCTACAA | TGCCCTGGGTATAACTCCCAA |
| CENPA | AAGACCACTTTGAGCAGTTGCCTG | ATGCTGCCACTAATGGTGAGGCTA |
| CENPH | CAGCCCCAGATGCAAGACG | GTGCAGTTGAAAGTCTCATCCT |
| CENPN | TGAACTGACAACAATCCTGAAGG | CTTGCACGCTTTTCCTCACAC |
| CENPW | AAGCCTCAACTTCGTCTGGAG | CACAAGCGTTTGTCCTGGACT |
| CEP128 | AATCATCCAGCGAATCAGATCAC | AGGAAGACTTCGAGTTCCCCT |
| CEP70 | AGAACAACGAGCTAATGACTTGG | AGCCCTACTTAGTGATTCATCCT |
| CNTRL | CAGATGAAAGCCCTTACATTGGC | CTGCCTGAGCACTGTCAATAAT |
| NCAPG | GAGGCTGCTGTCGATTAAGGA | AACTGTCTTATCATCCATCGTGC |
| NCAPH | GTCCTCGAAGACTTTCCTCAGA | TGAAATGTCAATACTCCTGCTGG |
| IL1R1 | ATGAAATTGATGTTCGTCCCTGT | ACCACGCAATAGTAATGTCCTG |
| LINC707 | CACTATGAGACTGGACTGGAACA | TGGTCTTGGGAGTGGTGC |
| LMNB1 | AAGCATGAAACGCGCTTGG | AGTTTGGCATGGTAAGTCTGC |
| SYNE2 | ACCACCCTATGGAAAGCTACT | CATCTCCCATCTGTCGAAGGC |
| NDC80 | GAGGACACGACAGTCACAATC | CTGGGGCCATGATGGATGC |
| UBE2C | TGGTCTGCCCTGTATGATGT | AAAAGCTGTGGGGTTTTTCC |
| UBE2L6 | TGGACGAGAACGGACAGATTT | GGCTCCCTGATATTCGGTCTATT |

**Supplementary table S2**

**List of up-regulated genes (log2FoldChange>1, pValue <0.05)**

| Gene Name | log2FoldChange | pValue |
| --- | --- | --- |
| TMEM119 | 5.187 | 0.00063 |
| C6orf141 | 4.1753 | 0.019218 |
| CENPA | 3.7759 | 2.36E-20 |
| CTHRC1 | 3.7157 | 4.84E-26 |
| E2F8 | 3.7141 | 0.000273 |
| LRRC34 | 3.3167 | 0.002348 |
| DDX12P | 3.278 | 0.000147 |
| RAD54L | 3.2734 | 1.28E-11 |
| BRCA2 | 3.267 | 7.78E-12 |
| TROAP | 3.1501 | 6.59E-25 |
| PRIM1 | 3.0776 | 0.000241 |
| PIF1 | 3.0595 | 2.53E-07 |
| GGT5 | 3.0508 | 9.82E-05 |
| CKAP2L | 3.0278 | 2.90E-34 |
| LINC00707 | 3.0247 | 0.025785 |
| ITPRIPL1 | 2.9724 | 0.000119 |
| VASH2 | 2.9536 | 0.007504 |
| CPXM1 | 2.9442 | 2.91E-31 |
| WISP1 | 2.918 | 0.009325 |
| ASPM | 2.8923 | 9.45E-76 |
| DMC1 | 2.877 | 0.049597 |
| NEIL3 | 2.8757 | 8.86E-09 |
| STAT5A | 2.871 | 0.010787 |
| KIF15 | 2.865 | 5.80E-15 |
| TTK | 2.8418 | 1.58E-25 |
| PLK4 | 2.8356 | 3.88E-20 |
| PLK1 | 2.8171 | 5.06E-58 |
| KIF20A | 2.8125 | 1.91E-47 |
| NIPAL4 | 2.8025 | 0.007523 |
| MKI67 | 2.7982 | 2.51E-105 |
| FES | 2.7959 | 0.00049 |
| DEPDC1 | 2.7844 | 3.08E-36 |
| MND1 | 2.7787 | 1.16E-05 |
| DEPDC1B | 2.7758 | 3.18E-12 |
| DLGAP5 | 2.7716 | 4.91E-48 |
| BUB1 | 2.7645 | 1.33E-45 |
| KIF2C | 2.7641 | 2.09E-41 |
| UBE2C | 2.7632 | 4.32E-45 |
| CEP128 | 2.7594 | 7.32E-11 |
| RP11-731J8.2 | 2.7355 | 0.026767 |
| CDC25C | 2.7174 | 1.64E-07 |
| HMGB2 | 2.7108 | 1.28E-65 |
| CDCA8 | 2.7011 | 3.97E-38 |
| SGOL1 | 2.6956 | 4.33E-07 |
| C11orf82 | 2.6867 | 2.35E-07 |
| ESCO2 | 2.6802 | 3.53E-08 |
| KIF18B | 2.677 | 1.09E-25 |
| BUB1B | 2.668 | 1.82E-25 |
| KIF14 | 2.667 | 1.09E-22 |
| FOXM1 | 2.6665 | 6.99E-53 |
| SAPCD2 | 2.6655 | 2.49E-09 |
| CDK1 | 2.6589 | 9.16E-39 |
| SPC25 | 2.6495 | 7.78E-12 |
| CLDN7 | 2.649 | 0.02624 |
| ABI3 | 2.6421 | 6.73E-05 |
| TOP2A | 2.6418 | 2.99E-105 |
| NCAPH | 2.6409 | 1.08E-26 |
| CDC20 | 2.6355 | 5.88E-63 |
| RAD51AP1 | 2.6308 | 6.18E-14 |
| PRC1 | 2.6307 | 7.08E-64 |
| WNT7B | 2.6283 | 0.001351 |
| SKA3 | 2.6245 | 1.86E-20 |
| LMNB1 | 2.6162 | 3.66E-50 |
| GFAP | 2.6108 | 0.032004 |
| SPAG5 | 2.6081 | 3.16E-39 |
| ORC1 | 2.607 | 2.59E-10 |
| NCAPG | 2.5913 | 6.41E-37 |
| NEK2 | 2.5906 | 9.93E-25 |
| CCDC152 | 2.5901 | 0.013468 |
| CTD-2510F5.4 | 2.5889 | 1.94E-09 |
| AC244102.1 | 2.5864 | 0.000598 |
| OIP5 | 2.5854 | 0.003761 |
| BIRC5 | 2.5783 | 2.41E-64 |
| MYBL2 | 2.573 | 3.17E-40 |
| POLQ | 2.5728 | 8.27E-15 |
| WDR62 | 2.5655 | 2.26E-19 |
| MCM10 | 2.5534 | 1.79E-15 |
| NEURL1B | 2.55 | 2.01E-06 |
| CBX2 | 2.5357 | 1.09E-09 |
| HMMR | 2.5315 | 2.10E-23 |
| C1orf106 | 2.5278 | 0.025199 |
| ERCC6L | 2.5141 | 6.50E-11 |
| KIF4A | 2.5096 | 2.62E-28 |
| BLM | 2.5092 | 1.07E-06 |
| CDKN3 | 2.5073 | 6.18E-14 |
| KIFC1 | 2.501 | 8.84E-37 |
| CENPF | 2.4981 | 2.67E-83 |
| NUSAP1 | 2.4956 | 2.77E-39 |
| GABRB1 | 2.4917 | 0.024309 |
| ZNF711 | 2.4912 | 5.95E-10 |
| ARHGAP11A | 2.4912 | 1.34E-52 |
| FAM83D | 2.4901 | 5.49E-25 |
| SYNE2 | 2.4895 | 1.17E-28 |
| DTL | 2.4882 | 3.24E-26 |
| PBK | 2.4814 | 7.36E-38 |
| RRM2 | 2.4798 | 1.94E-76 |
| KCNJ8 | 2.476 | 0.005445 |
| FAM64A | 2.4725 | 1.88E-17 |
| AURKB | 2.4654 | 2.07E-21 |
| TOX | 2.4504 | 0.000814 |
| GTSE1 | 2.4474 | 1.97E-34 |
| PRRX2 | 2.4399 | 0.000356 |
| CEP55 | 2.4397 | 1.96E-33 |
| MIS18BP1 | 2.4376 | 6.96E-11 |
| ASF1B | 2.4372 | 4.28E-11 |
| SKA1 | 2.4359 | 7.26E-11 |
| CENPE | 2.4311 | 2.38E-33 |
| KIF11 | 2.4274 | 2.26E-43 |
| IQGAP3 | 2.4117 | 3.14E-38 |
| CCNA2 | 2.4063 | 1.89E-39 |
| NUF2 | 2.4052 | 9.75E-29 |
| GAS2L3 | 2.4013 | 1.14E-15 |
| MYO7B | 2.398 | 2.64E-06 |
| HJURP | 2.3884 | 2.84E-25 |
| ZWINT | 2.3775 | 2.60E-46 |
| CASC5 | 2.3602 | 8.13E-28 |
| TMSB15A | 2.3572 | 1.03E-10 |
| CIT | 2.3566 | 4.70E-31 |
| SHCBP1 | 2.3547 | 4.01E-34 |
| CDCA7 | 2.3512 | 1.39E-18 |
| NDC80 | 2.338 | 7.16E-17 |
| SPC24 | 2.3306 | 1.32E-15 |
| SULT1C4 | 2.3173 | 0.000398 |
| FAM111B | 2.3121 | 7.01E-08 |
| FBXO5 | 2.3055 | 3.34E-17 |
| GSDMD | 2.2937 | 1.94E-09 |
| CCDC15 | 2.2912 | 1.93E-05 |
| CENPK | 2.2878 | 1.37E-12 |
| MAD2L1 | 2.2803 | 3.36E-33 |
| DCHS1 | 2.2644 | 5.87E-12 |
| PKMYT1 | 2.256 | 7.37E-15 |
| PALM3 | 2.2527 | 0.024449 |
| CDCA5 | 2.2526 | 8.06E-10 |
| ARHGAP11B | 2.2466 | 7.48E-12 |
| TRIM6 | 2.2368 | 3.85E-06 |
| FANCA | 2.2364 | 1.36E-17 |
| CENPM | 2.2299 | 3.90E-09 |
| WDR76 | 2.2172 | 1.06E-10 |
| KIF20B | 2.2171 | 2.49E-16 |
| MYOZ3 | 2.2037 | 0.042338 |
| AKR1C1 | 2.2 | 0.037297 |
| MLF1IP | 2.1937 | 4.82E-15 |
| SLC43A3 | 2.1899 | 1.25E-05 |
| SMC4 | 2.1861 | 4.24E-55 |
| TPX2 | 2.1764 | 1.49E-66 |
| CDKN2C | 2.1581 | 4.38E-18 |
| MELK | 2.1529 | 6.95E-30 |
| FAM129A | 2.1474 | 1.74E-09 |
| TCF19 | 2.1449 | 6.92E-30 |
| KIAA0101 | 2.1437 | 2.80E-19 |
| FANCI | 2.1428 | 3.54E-30 |
| CCNB2 | 2.1337 | 6.93E-26 |
| GIPC3 | 2.1236 | 0.002005 |
| EMILIN2 | 2.1207 | 8.23E-07 |
| KIF18A | 2.1193 | 1.37E-13 |
| FANCD2 | 2.1138 | 6.98E-18 |
| NPTX1 | 2.1114 | 2.21E-46 |
| TACC3 | 2.108 | 3.25E-31 |
| ADAMTS18 | 2.1021 | 1.16E-08 |
| BRCA1 | 2.1015 | 1.98E-14 |
| XRCC2 | 2.0972 | 1.16E-05 |
| HTRA3 | 2.0911 | 0.000171 |
| CDCA3 | 2.0855 | 5.57E-16 |
| MTFR2 | 2.0754 | 0.002198 |
| SLC22A3 | 2.075 | 0.017729 |
| ANLN | 2.0737 | 1.65E-63 |
| CCNB1 | 2.0729 | 4.24E-55 |
| KIF22 | 2.0706 | 3.08E-23 |
| LPAR3 | 2.0687 | 0.036523 |
| RP11-22B23.1 | 2.0665 | 0.002684 |
| ANXA10 | 2.0646 | 0.000751 |
| TRAIP | 2.0631 | 8.69E-06 |
| TK1 | 2.0578 | 5.24E-40 |
| PTTG1 | 2.0568 | 2.35E-15 |
| NLGN1 | 2.055 | 2.40E-08 |
| BRIP1 | 2.0523 | 9.76E-17 |
| APOBEC3B | 2.0481 | 7.28E-05 |
| ESPL1 | 2.0441 | 2.66E-13 |
| CDC45 | 2.0364 | 6.70E-11 |
| PTN | 2.0351 | 1.08E-16 |
| GUCY1B3 | 2.0167 | 0.001179 |
| PLEKHG4B | 2.0162 | 1.11E-05 |
| CENPI | 2.01 | 6.69E-11 |
| MDK | 2.0097 | 6.81E-52 |
| REC8 | 2.0008 | 0.000695 |
| TIFA | 1.9983 | 0.002154 |
| MXRA5 | 1.995 | 1.39E-51 |
| CCDC34 | 1.9943 | 4.73E-13 |
| BCHE | 1.9802 | 1.26E-05 |
| C3orf67 | 1.9755 | 0.007364 |
| KIAA1524 | 1.9697 | 3.40E-17 |
| RACGAP1 | 1.9669 | 6.20E-35 |
| LIN7A | 1.9658 | 8.26E-06 |
| CNTRL | 1.9658 | 1.36E-10 |
| GSG2 | 1.9637 | 0.001148 |
| IMPA2 | 1.9612 | 0.000244 |
| FBXO4 | 1.9576 | 0.001474 |
| RTKN2 | 1.9561 | 0.00016 |
| IGF2 | 1.9482 | 1.33E-09 |
| SPOCK3 | 1.9439 | 1.44E-05 |
| DSCC1 | 1.9352 | 4.56E-05 |
| CDH6 | 1.9332 | 2.46E-27 |
| C5orf34 | 1.9308 | 0.002699 |
| SERP2 | 1.93 | 0.006074 |
| GNG4 | 1.9268 | 0.001729 |
| HENMT1 | 1.9244 | 0.037474 |
| MCM7 | 1.9098 | 5.08E-51 |
| CLSPN | 1.9089 | 3.37E-10 |
| KIF24 | 1.9084 | 9.07E-05 |
| AIM1 | 1.8993 | 0.005867 |
| CRISPLD2 | 1.8988 | 0.000141 |
| TRIP13 | 1.8888 | 3.93E-19 |
| NCAPG2 | 1.8834 | 1.03E-33 |
| MAP2K6 | 1.8827 | 0.048776 |
| ARTN | 1.8801 | 0.006873 |
| ATAD5 | 1.8768 | 2.11E-05 |
| ZNF367 | 1.8684 | 0.000293 |
| SCIN | 1.8667 | 0.009993 |
| SULT1B1 | 1.8648 | 5.05E-05 |
| KIF23 | 1.8646 | 5.50E-19 |
| FAM161A | 1.8587 | 0.000174 |
| DPYSL3 | 1.8536 | 4.24E-39 |
| ZBED3 | 1.8508 | 0.004323 |
| GPSM2 | 1.8492 | 3.22E-11 |
| CDT1 | 1.8479 | 1.34E-12 |
| DBF4 | 1.8477 | 5.75E-12 |
| SGOL2 | 1.8447 | 1.33E-14 |
| RP11-89K11.1 | 1.8415 | 0.046243 |
| HNMT | 1.8359 | 0.036813 |
| PARP9 | 1.8343 | 5.86E-07 |
| VILL | 1.8317 | 0.006693 |
| PCDH18 | 1.8297 | 2.60E-12 |
| GINS3 | 1.8254 | 0.001028 |
| KNTC1 | 1.8223 | 4.38E-18 |
| CSF1R | 1.8186 | 0.011223 |
| CXCL5 | 1.8166 | 0.03457 |
| EME1 | 1.8144 | 0.001744 |
| CDCA2 | 1.8129 | 3.79E-12 |
| RHOU | 1.8 | 4.41E-09 |
| TMPO | 1.7977 | 1.08E-36 |
| TIMELESS | 1.7975 | 4.51E-25 |
| PRTFDC1 | 1.7939 | 5.07E-11 |
| TET1 | 1.7928 | 0.024265 |
| PLA2G4A | 1.7908 | 0.0026 |
| HHEX | 1.7869 | 2.44E-08 |
| BORA | 1.7864 | 0.010746 |
| PKN3 | 1.781 | 1.91E-06 |
| CENPH | 1.7775 | 1.40E-07 |
| RBL1 | 1.775 | 3.66E-09 |
| PTX3 | 1.7744 | 3.05E-28 |
| HGF | 1.7697 | 0.016095 |
| CENPW | 1.7627 | 1.93E-08 |
| IFI35 | 1.7619 | 0.006092 |
| SAMD11 | 1.7606 | 1.94E-05 |
| SCARA3 | 1.7563 | 9.34E-34 |
| EZH2 | 1.7545 | 7.68E-15 |
| MCM5 | 1.7513 | 6.31E-30 |
| RELN | 1.7512 | 6.32E-13 |
| ST6GALNAC3 | 1.7503 | 0.001263 |
| CHAF1B | 1.7415 | 1.17E-09 |
| SERPINB9 | 1.7414 | 0.000952 |
| PSMC3IP | 1.7411 | 0.000913 |
| MTBP | 1.7337 | 0.004591 |
| BZRAP1 | 1.728 | 0.0046 |
| POLE2 | 1.7278 | 0.001599 |
| FAM111A | 1.7265 | 1.13E-08 |
| GMNN | 1.7212 | 5.84E-14 |
| MBNL3 | 1.7188 | 0.011207 |
| DBF4B | 1.7178 | 1.47E-08 |
| STEAP1B | 1.717 | 0.001518 |
| BTN3A1 | 1.7023 | 9.03E-06 |
| RFC3 | 1.7004 | 5.79E-11 |
| MCM2 | 1.696 | 4.65E-19 |
| PTGES | 1.6953 | 2.92E-05 |
| CDC6 | 1.6951 | 7.83E-15 |
| CXCL6 | 1.6896 | 0.000617 |
| CXCL1 | 1.679 | 1.79E-08 |
| SPON2 | 1.6722 | 7.46E-06 |
| RAD54B | 1.671 | 0.019428 |
| FKBP5 | 1.6599 | 4.58E-07 |
| CCDC138 | 1.6553 | 0.003126 |
| HELLS | 1.6543 | 1.06E-10 |
| STIL | 1.6455 | 5.38E-11 |
| POC1A | 1.6369 | 2.15E-09 |
| FGFR3 | 1.6364 | 0.020864 |
| COL9A2 | 1.6311 | 7.50E-05 |
| NR5A2 | 1.6305 | 5.76E-05 |
| CEP152 | 1.6284 | 2.44E-05 |
| RNASEH2A | 1.6245 | 2.64E-15 |
| KIAA1211 | 1.62 | 0.000509 |
| EXO1 | 1.6181 | 1.44E-06 |
| TGFB3 | 1.6169 | 0.003071 |
| GPC2 | 1.6163 | 0.004367 |
| DEK | 1.6151 | 4.49E-37 |
| SLFN11 | 1.6103 | 1.83E-13 |
| TMEM194B | 1.6084 | 0.006264 |
| C4orf21 | 1.6066 | 0.00028 |
| MIS18A | 1.604 | 6.64E-07 |
| GINS1 | 1.6038 | 9.68E-09 |
| GINS2 | 1.6026 | 2.82E-09 |
| H1F0 | 1.5956 | 1.25E-39 |
| WHSC1 | 1.5946 | 3.58E-38 |
| PPFIA2 | 1.5915 | 0.017913 |
| CCDC18 | 1.5879 | 7.44E-05 |
| RCOR2 | 1.5849 | 0.000548 |
| TICRR | 1.572 | 1.87E-05 |
| RPL39L | 1.566 | 1.94E-06 |
| ATAD2 | 1.5652 | 5.23E-18 |
| CENPN | 1.5544 | 3.34E-08 |
| SLC37A2 | 1.5527 | 2.12E-13 |
| NCAPD2 | 1.5511 | 3.77E-34 |
| ITPR3 | 1.551 | 5.29E-06 |
| PSRC1 | 1.5509 | 1.09E-10 |
| C18orf54 | 1.545 | 4.18E-08 |
| C1orf112 | 1.5412 | 1.34E-05 |
| GAL3ST4 | 1.5387 | 0.034548 |
| CYYR1 | 1.5385 | 0.00064 |
| ADAM33 | 1.5368 | 0.000754 |
| TFAP4 | 1.5287 | 0.001381 |
| SETD6 | 1.5277 | 0.001384 |
| GNG2 | 1.5277 | 2.08E-12 |
| EPCAM | 1.5274 | 0.030194 |
| PRR11 | 1.5227 | 2.75E-17 |
| CRISPLD1 | 1.5205 | 8.10E-05 |
| TRIM14 | 1.511 | 7.70E-10 |
| MTMR9LP | 1.51 | 0.021906 |
| CHAF1A | 1.5096 | 4.61E-16 |
| ALPK1 | 1.5089 | 0.039611 |
| MIR155HG | 1.5072 | 0.043734 |
| RP11-713C5.1 | 1.5069 | 0.043734 |
| NYNRIN | 1.5058 | 1.26E-16 |
| H2AFZ | 1.5023 | 3.76E-41 |
| GREM2 | 1.4995 | 0.010116 |
| ESM1 | 1.4993 | 0.000751 |
| PHF19 | 1.4991 | 1.40E-20 |
| CHN1 | 1.4991 | 0.000549 |
| CORO1A | 1.4972 | 0.005785 |
| PARPBP | 1.4944 | 0.000166 |
| RP11-673C5.1 | 1.4925 | 1.58E-07 |
| SECTM1 | 1.4906 | 0.034508 |
| ANGPT2 | 1.4903 | 2.40E-06 |
| DCLRE1B | 1.4832 | 3.89E-06 |
| ETV4 | 1.4815 | 1.37E-09 |
| CKAP2 | 1.479 | 1.12E-23 |
| CENPJ | 1.4761 | 1.19E-08 |
| FANCG | 1.4756 | 1.21E-08 |
| IL1R1 | 1.4733 | 1.17E-17 |
| COL12A1 | 1.4729 | 1.15E-30 |
| UBE2T | 1.4649 | 0.000152 |
| MATN3 | 1.4597 | 1.04E-07 |
| ARHGAP19 | 1.4515 | 9.66E-08 |
| WDHD1 | 1.4493 | 3.91E-10 |
| MMS22L | 1.4462 | 1.07E-07 |
| DKK1 | 1.4452 | 3.68E-13 |
| PEX5L | 1.4347 | 0.002056 |
| TYMS | 1.4323 | 2.32E-08 |
| HIRIP3 | 1.4277 | 1.73E-06 |
| POLD3 | 1.4276 | 1.37E-08 |
| CHEK1 | 1.4275 | 7.70E-09 |
| C17orf53 | 1.4226 | 0.007376 |
| SHMT1 | 1.4219 | 7.75E-05 |
| RTTN | 1.41 | 0.000393 |
| SLC12A8 | 1.4034 | 0.000715 |
| ORC6 | 1.3961 | 1.34E-07 |
| LIN9 | 1.3953 | 0.001124 |
| COL6A3 | 1.3911 | 3.77E-34 |
| PSIP1 | 1.3904 | 1.67E-19 |
| LXN | 1.3883 | 2.25E-06 |
| POLA1 | 1.3863 | 1.85E-11 |
| CCNF | 1.3856 | 3.94E-10 |
| DHFR | 1.3819 | 2.69E-18 |
| RASGRF2 | 1.379 | 1.56E-05 |
| HMGB3 | 1.3735 | 4.37E-19 |
| FEN1 | 1.373 | 3.15E-10 |
| GEM | 1.3712 | 1.34E-11 |
| DTX3L | 1.3708 | 8.77E-06 |
| HAS2 | 1.3671 | 0.000128 |
| HMGB1 | 1.3633 | 1.47E-42 |
| CPS1 | 1.3593 | 1.52E-06 |
| BARD1 | 1.3563 | 0.000344 |
| USP1 | 1.3561 | 2.71E-16 |
| APOL6 | 1.355 | 0.032491 |
| MCM3 | 1.3531 | 3.32E-20 |
| STEAP1 | 1.3512 | 0.000206 |
| RFC4 | 1.3496 | 0.00585 |
| RMI2 | 1.3433 | 0.000388 |
| DOCK10 | 1.3396 | 1.20E-13 |
| HIC1 | 1.3393 | 3.26E-16 |
| BTN3A3 | 1.3382 | 0.042047 |
| ALX1 | 1.3381 | 0.010861 |
| SOCS2 | 1.3355 | 1.10E-08 |
| ZNF785 | 1.3354 | 0.04207 |
| INCENP | 1.3349 | 2.35E-15 |
| PLSCR1 | 1.3317 | 0.000122 |
| MCM6 | 1.3306 | 1.29E-14 |
| PHGDH | 1.3288 | 7.78E-12 |
| GPC6 | 1.3275 | 0.002222 |
| ARNTL2 | 1.3271 | 1.30E-10 |
| CDC25B | 1.3255 | 3.00E-22 |
| ETV1 | 1.3204 | 5.79E-16 |
| KISS1 | 1.3204 | 5.87E-08 |
| STMN1 | 1.3202 | 1.85E-36 |
| CKS2 | 1.3191 | 7.44E-15 |
| HHIPL1 | 1.3186 | 2.01E-06 |
| KLHL23 | 1.3145 | 0.008838 |
| FUT4 | 1.3075 | 0.026752 |
| VRK1 | 1.3031 | 3.05E-07 |
| CCDC109B | 1.3009 | 1.08E-06 |
| FAIM | 1.2988 | 0.00247 |
| RPL22L1 | 1.291 | 9.95E-07 |
| PAQR8 | 1.2907 | 0.001661 |
| NR2C2AP | 1.2892 | 0.015568 |
| MAOA | 1.2891 | 0.019472 |
| NMI | 1.287 | 0.026829 |
| RAD51 | 1.276 | 0.002399 |
| SPIN4 | 1.2748 | 0.002098 |
| TRAF1 | 1.2739 | 0.001788 |
| ANXA3 | 1.2735 | 2.74E-06 |
| GINS4 | 1.2704 | 7.84E-05 |
| TNNT1 | 1.2699 | 0.003059 |
| PATZ1 | 1.2694 | 2.41E-06 |
| RRM1 | 1.2667 | 3.29E-23 |
| NOX4 | 1.2646 | 0.000271 |
| DNA2 | 1.2624 | 0.048212 |
| CEP70 | 1.2576 | 0.000574 |
| TSPY26P | 1.2564 | 2.88E-05 |
| DUT | 1.2555 | 2.73E-11 |
| PDE3A | 1.2546 | 0.005467 |
| MCM8 | 1.2532 | 2.53E-07 |
| BTN3A2 | 1.2504 | 0.000173 |
| CDC7 | 1.2499 | 7.66E-06 |
| RP11-166D19.1 | 1.2494 | 1.66E-21 |
| AURKA | 1.24 | 3.89E-17 |
| COL7A1 | 1.2382 | 2.20E-17 |
| GPX7 | 1.2377 | 0.010932 |
| MASTL | 1.2373 | 1.33E-05 |
| ME3 | 1.2338 | 2.43E-05 |
| PASK | 1.2337 | 0.002269 |
| PROB1 | 1.2332 | 0.00135 |
| GABRA3 | 1.2321 | 4.24E-05 |
| SLIT2 | 1.2314 | 5.15E-13 |
| CDCA7L | 1.2311 | 1.06E-05 |
| NASP | 1.2301 | 2.09E-21 |
| ZNF486 | 1.2286 | 0.0046 |
| LAMA2 | 1.2247 | 0.001969 |
| EMP2 | 1.2228 | 0.000703 |
| CXCL12 | 1.2226 | 1.60E-28 |
| HSPBAP1 | 1.2191 | 0.048764 |
| DDX60 | 1.2139 | 0.014332 |
| RNF144A | 1.2127 | 0.000117 |
| CLDN11 | 1.2124 | 1.63E-23 |
| NCAPD3 | 1.206 | 7.46E-13 |
| IGDCC4 | 1.2027 | 0.000114 |
| SOD3 | 1.2027 | 9.95E-05 |
| PRRT2 | 1.1978 | 0.002841 |
| PKIA | 1.1934 | 3.55E-09 |
| GPR56 | 1.1911 | 1.48E-15 |
| H2AFX | 1.1906 | 3.08E-20 |
| SSPN | 1.1905 | 8.43E-07 |
| HAUS8 | 1.1879 | 0.034965 |
| SMC2 | 1.1876 | 1.47E-14 |
| KIAA1841 | 1.1866 | 0.000561 |
| MCM4 | 1.1824 | 1.69E-19 |
| LIG1 | 1.1817 | 1.10E-09 |
| TOPBP1 | 1.1794 | 7.91E-12 |
| ACSS3 | 1.1776 | 0.000297 |
| METTL4 | 1.1775 | 0.000839 |
| C16orf59 | 1.1725 | 0.001476 |
| NINL | 1.1701 | 9.76E-06 |
| COL16A1 | 1.1693 | 1.60E-09 |
| TIPIN | 1.1692 | 0.002637 |
| CHTF18 | 1.1692 | 3.27E-07 |
| TMEM48 | 1.1691 | 1.64E-10 |
| F2RL1 | 1.1684 | 9.80E-12 |
| RGS17 | 1.1668 | 0.002171 |
| CDC25A | 1.1648 | 3.75E-06 |
| CKS1B | 1.1626 | 2.17E-14 |
| ALKBH2 | 1.1622 | 0.007692 |
| EVA1C | 1.156 | 0.016307 |
| ZC3HAV1 | 1.154 | 2.60E-08 |
| CENPO | 1.1498 | 2.57E-06 |
| ECT2 | 1.147 | 1.18E-14 |
| DDX39A | 1.1434 | 5.43E-17 |
| GEN1 | 1.1425 | 3.11E-05 |
| NRGN | 1.1401 | 3.76E-05 |
| SLC7A11 | 1.1355 | 2.07E-22 |
| CYB5R2 | 1.1308 | 0.006514 |
| LIMD2 | 1.1296 | 2.44E-10 |
| PRDM8 | 1.1254 | 3.08E-15 |
| CECR1 | 1.1253 | 1.59E-10 |
| MAP3K5 | 1.1194 | 2.66E-06 |
| PARP14 | 1.1168 | 8.40E-07 |
| EXOSC8 | 1.1157 | 4.09E-07 |
| BMF | 1.1135 | 5.88E-05 |
| DLG3 | 1.1117 | 1.95E-07 |
| ANKRD32 | 1.1094 | 0.006468 |
| ZNF22 | 1.1079 | 0.000443 |
| HMGN3 | 1.1043 | 0.00591 |
| FANCE | 1.1035 | 0.012795 |
| ARHGEF6 | 1.1022 | 0.000513 |
| NRAS | 1.0988 | 5.79E-19 |
| SV2A | 1.0978 | 3.00E-12 |
| KIAA1009 | 1.0917 | 0.030075 |
| NLRC5 | 1.0879 | 0.001906 |
| PARP12 | 1.0867 | 0.01794 |
| CDK2 | 1.0866 | 5.64E-07 |
| FBLN1 | 1.0854 | 2.84E-21 |
| NRP2 | 1.0832 | 1.79E-16 |
| ZNF184 | 1.0826 | 0.01133 |
| SLC7A2 | 1.076 | 0.03701 |
| ITGA10 | 1.0744 | 1.73E-08 |
| RFC5 | 1.0738 | 0.000576 |
| GNB3 | 1.0688 | 0.024009 |
| DOCK11 | 1.0685 | 8.61E-07 |
| ARAP3 | 1.0681 | 1.34E-11 |
| MAPK13 | 1.0673 | 0.000121 |
| TWIST2 | 1.0642 | 4.90E-06 |
| NRM | 1.0624 | 7.51E-06 |
| RHNO1 | 1.0611 | 0.000101 |
| SMC3 | 1.0569 | 1.95E-07 |
| TBX3 | 1.0565 | 6.48E-06 |
| LRRC49 | 1.0549 | 0.010856 |
| EXOC6 | 1.0538 | 0.006693 |
| DNMT1 | 1.0534 | 5.29E-20 |
| PLCL2 | 1.0532 | 0.007865 |
| PRPS2 | 1.0518 | 4.08E-05 |
| MXD3 | 1.0517 | 0.013107 |
| SUV39H1 | 1.0512 | 0.000903 |
| NUP107 | 1.0485 | 1.03E-08 |
| ITGB3BP | 1.0431 | 0.000828 |
| RBBP8 | 1.0428 | 6.52E-06 |
| TNFRSF19 | 1.0414 | 2.88E-05 |
| CC2D2A | 1.0409 | 2.37E-07 |
| UBE2L6 | 1.0395 | 0.000251 |
| STON1 | 1.0375 | 0.001856 |
| DNALI1 | 1.0362 | 0.001091 |
| DIS3L | 1.0317 | 1.14E-05 |
| PRDM16 | 1.0305 | 0.00114 |
| KIAA1755 | 1.0273 | 1.47E-06 |
| CENPC1 | 1.0225 | 0.002848 |
| KPNA2 | 1.0221 | 2.56E-22 |
| HMGN2 | 1.0221 | 3.09E-23 |
| N4BP2 | 1.0211 | 0.003532 |
| FAM213A | 1.0209 | 4.48E-07 |
| TRANK1 | 1.0209 | 7.30E-06 |
| MYRF | 1.02 | 0.000113 |
| GLI3 | 1.0193 | 8.26E-05 |
| FAM167A | 1.0162 | 4.45E-07 |
| LRRC8C | 1.0161 | 7.91E-07 |
| MECOM | 1.0139 | 1.75E-05 |
| C4orf46 | 1.0114 | 5.45E-05 |
| PLEKHA4 | 1.0085 | 0.001264 |
| PGBD1 | 1.0022 | 0.000606 |
| SYT1 | 1.001 | 0.000153 |

**Supplementary table S3**

**List of down-regulated gene (log2FoldChange<1, pValue<0.05)**

| Gene Name | log2FoldChange | pValue |
| --- | --- | --- |
| IGSF23 | -4.9372 | 0.0019715 |
| VAV3 | -4.508 | 2.61E-07 |
| MIR210HG | -4.3966 | 5.19E-17 |
| PADI1 | -3.6755 | 0.02669 |
| AK4 | -3.5099 | 1.04E-55 |
| CMAHP | -3.4809 | 0.00080376 |
| KCTD4 | -3.441 | 5.95E-08 |
| RPL17P50 | -3.3941 | 0.0015245 |
| MYOZ2 | -2.8679 | 0.012532 |
| AC003102.3 | -2.7055 | 0.00046177 |
| ACTC1 | -2.6347 | 1.95E-07 |
| DUSP27 | -2.615 | 0.0098392 |
| ARPP21 | -2.5781 | 0.002286 |
| TMEM151A | -2.5324 | 4.19E-05 |
| OR52K3P | -2.4954 | 0.00064776 |
| TENM2 | -2.4649 | 0.00075121 |
| ANKRD1 | -2.435 | 2.22E-50 |
| TMEM40 | -2.4144 | 8.70E-15 |
| KCNC3 | -2.3597 | 0.00031899 |
| PTPRR | -2.222 | 0.01417 |
| NLRP10 | -2.2216 | 0.0042856 |
| RP11-267A15.1 | -2.2151 | 0.0043405 |
| CNNM1 | -2.2109 | 0.032431 |
| GDF6 | -2.2106 | 1.01E-06 |
| CCND2 | -2.166 | 4.72E-36 |
| ADM2 | -2.1578 | 0.049101 |
| PPFIA4 | -2.1521 | 2.92E-05 |
| JAG2 | -2.1354 | 1.31E-07 |
| GSN-AS1 | -2.1354 | 0.011466 |
| NFASC | -2.0921 | 4.56E-06 |
| RP11-163F15.1 | -2.0742 | 0.01179 |
| HAPLN1 | -2.0702 | 2.67E-83 |
| TLL2 | -2.0397 | 2.10E-12 |
| EEF1A2 | -2.0279 | 1.65E-07 |
| ADRA2C | -2.019 | 0.00012355 |
| ANGPTL4 | -2.0137 | 9.09E-78 |
| FBXL16 | -2.0027 | 2.00E-13 |
| MMP24 | -1.9934 | 9.24E-09 |
| IL6 | -1.9838 | 0.0054465 |
| USP2 | -1.9649 | 0.045681 |
| MFAP5 | -1.9482 | 0.022482 |
| GNG7 | -1.8731 | 0.029684 |
| PCSK1N | -1.8639 | 1.87E-10 |
| HIST1H3D | -1.8224 | 0.033014 |
| RGS5 | -1.8191 | 2.22E-74 |
| HAS3 | -1.8056 | 5.64E-16 |
| CRYAB | -1.7936 | 8.43E-31 |
| C7orf10 | -1.7784 | 0.0066495 |
| CLDN1 | -1.7757 | 1.83E-48 |
| ARRDC4 | -1.7754 | 6.04E-17 |
| HK2 | -1.7703 | 3.69E-20 |
| CREB5 | -1.7702 | 1.79E-16 |
| SFTA1P | -1.7629 | 0.0013539 |
| WBSCR17 | -1.7546 | 0.0093254 |
| RP11-420A23.1 | -1.7411 | 0.0044909 |
| KRTAP2-3 | -1.7348 | 2.12E-18 |
| ACHE | -1.7307 | 8.27E-05 |
| TMEM178B | -1.7224 | 9.96E-05 |
| ROR1 | -1.709 | 3.45E-07 |
| NPAS1 | -1.6958 | 5.11E-09 |
| RP11-10C24.3 | -1.6934 | 0.00020818 |
| NXPH4 | -1.6875 | 0.00064324 |
| CD36 | -1.6683 | 0.010364 |
| P4HA1 | -1.6669 | 2.48E-54 |
| NRXN3 | -1.6504 | 0.014731 |
| CERS1 | -1.6478 | 0.011253 |
| MALAT1 | -1.6468 | 1.56E-35 |
| PTHLH | -1.6417 | 0.00032546 |
| ADAMTS5 | -1.6351 | 3.05E-08 |
| NUDT14 | -1.6283 | 0.016486 |
| MAP3K7CL | -1.6256 | 3.99E-07 |
| GPRIN3 | -1.6198 | 0.046966 |
| ASS1 | -1.6164 | 1.91E-20 |
| SERPINE2 | -1.5984 | 1.19E-62 |
| CADM2 | -1.5851 | 0.02313 |
| RP11-224O19.2 | -1.5689 | 1.59E-08 |
| SERPINB7 | -1.5529 | 2.39E-18 |
| CPA4 | -1.5495 | 3.75E-58 |
| KIT | -1.5452 | 0.00074946 |
| PRPS1 | -1.5448 | 2.08E-48 |
| CLSTN2 | -1.5324 | 1.83E-06 |
| CELF2 | -1.5242 | 0.00061714 |
| AC012360.6 | -1.5134 | 0.0069626 |
| KNDC1 | -1.5082 | 0.019596 |
| TXNIP | -1.5037 | 4.15E-36 |
| LDB3 | -1.489 | 0.023131 |
| IL1RAPL1 | -1.488 | 0.0012248 |
| ZNF219 | -1.4826 | 3.98E-05 |
| UNC13A | -1.481 | 4.02E-06 |
| SERINC2 | -1.4789 | 7.33E-05 |
| TUFT1 | -1.4752 | 2.44E-20 |
| LINC00607 | -1.4651 | 0.045607 |
| SLC1A1 | -1.4649 | 5.11E-11 |
| AL450307.1 | -1.4615 | 1.06E-21 |
| RNF128 | -1.4606 | 0.00015359 |
| JPH2 | -1.4396 | 0.034027 |
| CTD-2021J15.2 | -1.4337 | 1.18E-06 |
| MMP15 | -1.4327 | 5.79E-07 |
| TMEM59L | -1.4305 | 4.04E-08 |
| PNMA2 | -1.4123 | 0.00025725 |
| HIST1H4H | -1.4111 | 0.0014852 |
| GJA5 | -1.4111 | 0.044556 |
| GALNT3 | -1.4047 | 1.33E-05 |
| RIMS2 | -1.3983 | 0.0021928 |
| LRRN1 | -1.3552 | 1.23E-14 |
| FAM212B | -1.354 | 3.48E-09 |
| GPR1 | -1.3204 | 0.0024225 |
| ALS2CL | -1.3191 | 2.11E-05 |
| ADAMTS1 | -1.3184 | 5.93E-31 |
| FRMPD3 | -1.3168 | 0.022385 |
| HIST1H2BC | -1.3145 | 8.58E-06 |
| RP11-879F14.2 | -1.3051 | 3.50E-08 |
| TTC18 | -1.2912 | 0.041733 |
| HBEGF | -1.2869 | 1.98E-23 |
| SLC35F3 | -1.2843 | 0.025309 |
| STYK1 | -1.2792 | 0.0027173 |
| FAM83H | -1.275 | 0.00050909 |
| BTG2 | -1.2746 | 1.43E-12 |
| SIRPA | -1.2727 | 4.18E-31 |
| SERPINE1 | -1.268 | 3.36E-45 |
| RASSF7 | -1.2648 | 6.61E-07 |
| GRIA3 | -1.2587 | 0.00027143 |
| KCTD20 | -1.2566 | 4.32E-35 |
| LMCD1 | -1.2508 | 1.14E-08 |
| L1CAM | -1.2491 | 8.07E-21 |
| FAM115C | -1.237 | 0.0034585 |
| MAMDC2 | -1.2346 | 1.43E-31 |
| NRG1 | -1.2245 | 1.16E-16 |
| MOK | -1.2156 | 1.98E-26 |
| ULBP2 | -1.2038 | 1.87E-07 |
| PCDH10 | -1.2018 | 1.39E-12 |
| HIST1H1C | -1.2013 | 7.90E-08 |
| CLCA2 | -1.2002 | 2.06E-06 |
| HIST2H2BE | -1.1997 | 4.16E-14 |
| MAP3K9 | -1.1993 | 0.037285 |
| HIST1H2BK | -1.1983 | 9.79E-09 |
| DUSP8 | -1.1954 | 5.11E-05 |
| PVR | -1.1914 | 3.82E-30 |
| COL11A1 | -1.1877 | 9.36E-15 |
| KCNK6 | -1.17 | 2.96E-09 |
| SYNGR2 | -1.1609 | 3.82E-15 |
| SELPLG | -1.1412 | 0.0002368 |
| IGFBP7 | -1.1399 | 2.52E-09 |
| THBS1 | -1.1345 | 1.15E-30 |
| IRGQ | -1.1341 | 3.08E-14 |
| RP11-758N13.1 | -1.1302 | 0.042299 |
| LIPG | -1.1285 | 5.76E-05 |
| HIST1H2AC | -1.1272 | 1.00E-16 |
| SCD5 | -1.1248 | 1.85E-21 |
| ABAT | -1.1218 | 1.97E-07 |
| TGFB2 | -1.1186 | 9.96E-26 |
| EBI3 | -1.1182 | 0.0086495 |
| BNIP3 | -1.1162 | 2.07E-06 |
| CCDC113 | -1.1155 | 0.041998 |
| P4HA2 | -1.1144 | 5.09E-26 |
| DPP4 | -1.1076 | 1.31E-14 |
| PDK1 | -1.0985 | 8.42E-10 |
| GAMT | -1.0953 | 4.01E-11 |
| IGFBP3 | -1.0931 | 1.46E-22 |
| EXTL1 | -1.092 | 2.01E-15 |
| KRT18 | -1.0869 | 1.42E-14 |
| KLHL13 | -1.0861 | 0.033833 |
| PLAT | -1.0797 | 3.11E-23 |
| MN1 | -1.0788 | 1.09E-05 |
| LIMS2 | -1.0741 | 0.00014619 |
| PROSER2 | -1.0732 | 0.015086 |
| RP11-366L20.2 | -1.07 | 0.0012336 |
| CST1 | -1.0687 | 0.0022287 |
| SORT1 | -1.062 | 3.38E-17 |
| PTPRB | -1.0596 | 0.00012654 |
| NF2 | -1.0569 | 2.15E-21 |
| RP11-54A9.1 | -1.0549 | 0.0070751 |
| DYSF | -1.0537 | 7.34E-11 |
| FAM198B | -1.0537 | 0.0011757 |
| HS3ST1 | -1.0521 | 0.0017177 |
| NDRG1 | -1.0505 | 8.65E-15 |
| CDK15 | -1.0487 | 0.038765 |
| FADS3 | -1.0427 | 5.52E-12 |
| DNASE1L1 | -1.0309 | 6.04E-05 |
| TTC3P1 | -1.0277 | 0.00089101 |
| RCAN1 | -1.024 | 2.39E-22 |
| FBXO2 | -1.021 | 6.29E-07 |
| EFHD1 | -1.0196 | 0.00061536 |
| RAB3A | -1.0144 | 0.0099778 |
| GPR155 | -1.0143 | 1.68E-05 |
| QPCTL | -1.0076 | 9.48E-09 |
| SVEP1 | -1.0054 | 4.90E-06 |
| EDN1 | -1.0008 | 1.69E-09 |
